# Late Infusion of Cloned Marrow Fibroblasts Stimulates Endogenous Recovery from Radiation-Induced Lung Injury

**DOI:** 10.1371/journal.pone.0057179

**Published:** 2013-03-08

**Authors:** Mineo Iwata, David K. Madtes, Kraig Abrams, Wayne J. E. Lamm, Robb W. Glenny, Richard A. Nash, Aravind Ramakrishnan, Beverly Torok-Storb

**Affiliations:** 1 Clinical Research Division, Fred Hutchinson Cancer Research Center, Seattle, Washington, United States of America; 2 Department of Medicine, University of Washington, Seattle, Washington, United States of America; 3 Department of Physiology and Biophysics, University of Washington, Seattle, Washington, United States of America; University of Illinois at Chicago, United States of America

## Abstract

In the current study, we used a canine model of radiation-induced lung injury to test the effect of a single i.v. infusion of 10×10^6^/kg of marrow fibroblasts on the progression of damage following 15 Gy exposure to the right lung. The fibroblasts, designated DS1 cells, are a cloned population of immortalized cells isolated from a primary culture of marrow stromal cells. DS1 cells were infused at week 5 post-irradiation when lung damage was evident by imaging with high-resolution computed tomography (CT). At 13 weeks post-irradiation we found that 4 out of 5 dogs receiving DS1 cells had significantly improved pulmonary function compared to 0 out of 5 control dogs (p = 0.047, Fisher’s Exact). Pulmonary function was measured as the single breath diffusion capacity-hematocrit (DLCO-Hct), the total inspiratory capacity (IC), and the total lung capacity (TLC), which differed significantly between control and DS1-treated dogs; p = 0.002, p = 0.005, and p = 0.004, respectively. The DS1-treated dogs also had less pneumonitis detected by CT imaging and an increased number of TTF-1 (thyroid transcription factor 1, NKX2-1) positive cells in the bronchioli and alveoli compared to control dogs. Endothelial-like progenitor cells (ELC) of host origin, detected by colony assays, were found in peripheral blood after DS1 cell infusion. ELC numbers peaked one day after infusion, and were not detectable by 7 days. These data suggest that infusion of marrow fibroblasts stimulates mobilization of ELC, which is associated with a reduction in otherwise progressive radiation-induced lung injury. We hypothesize that these two observations are related, specifically that circulating ELC contribute to increased angiogenesis, which facilitates endogenous lung repair.

## Introduction

The lung is a highly complex, three-dimensional structure comprised of more than 40 distinct cell types organized into conducting airways and vasculature which terminate in the distal alveolar-capillary units [1], [2]. Non-lethal lung injury activates organized repair mechanisms to repopulate areas of damage with appropriate cell types needed to restore function [3]. Human and murine studies suggest that both bone marrow-derived cells and resident lung cells contribute to the lung repair process following injury [4]–[7]. Interplay between lung stem cells and pulmonary capillary endothelial cells has been proposed to induce regenerative lung alveolarization [8]. Unfortunately, in lung diseases such as radiation pneumonitis, lung allograft rejection, and acute respiratory distress syndrome, the alveolar disruption and vascular loss are often extensive and progressive with limited spontaneous regeneration of normal lung architecture.Although there are currently some medical countermeasures for the management of hematopoietic injury after radiation exposure [9], [10], there are no effective countermeasures against radiation-induced pneumonitis.

Vascular damage is known to follow radiation exposure [11]. Vascular repair is most likely accomplished through new vessel formation by sprouting angiogenesis, by endothelial cells from nearby vessels, and/or by circulating endothelial progenitor cells postulated to originate in the bone marrow [8], [12], [13]. These circulating progenitors may be directly incorporated into areas of vascular damage and/or function to modify the local microenvironment to facilitate regeneration by endogenous lung repopulating cells.

In this study, we found that a single infusion of a homogeneous population of canine marrow-derived fibroblasts (DS1 cells) into dogs with radiation-induced pneumonitis was followed by increased numbers of endogenous endothelial progenitor-like cells (ELC) in the peripheral blood. Four out of five of the DS1-infused dogs showed improvement in pulmonary function after one injection compared to none of the irradiated, untreated control dogs.

## Materials and Methods

### Dogs

All dogs were either purpose-bred by Fred Hutchinson Cancer Research Center (FHCRC) or obtained from Class “A” vendors. Breeds used included beagles or beagle crosses mixed with mini-mongrels, hounds, or golden retrievers. Canine demographics are described in Table S1. Experiments were conducted according to the principles outlined in the Guide for Laboratory Animal Facilities and Care prepared by the National Academy of Sciences, National Research Council. The Institutional Animal Care and Use Committee of Fred Hutchinson Cancer Research Center (FHCRC) approved the research protocol and the American Association of Accreditation of Laboratory Animal Care certified the kennels.

### Lung Irradiation

Right lung irradiation was performed on 10 dogs as described in *Methods S1*. Eighty percent of the right lung was irradiated with a dose of 5 Gy at 0.07 Gy/minute, followed by a radiation boost of 10 Gy at 5 Gy/minute to the same location. The experimental design of the lung irradiation as well as pulmonary function tests (PFTs) and computed tomography (CT) scans are summarized in Figure S4. Five dogs were infused with DS1 cells at 5 weeks post radiation, and 5 dogs were described as irradiated-untreated controls.

### Cells

Canine marrow-derived fibroblasts, designated as DS1 cells, were used in this study. These cells were generated from primary cultures of marrow stroma immortalized with a retrovirus containing the human papilloma virus E6/E7 genes [14]. The cells were cultured in RPMI1640 medium containing 10% fetal calf serum, L-glutamine (0.4 mg/mL), sodium pyruvate (1 mmol/L), penicillin (100 U/mL) and streptomycin sulfate (100 pg/mL).Prior to infusion, DS1 cells were harvested using trypsin, washed with Hank’s Balanced Salt Solution (HBSS) and filtered through a cell strainer (BD Falcon, Bedford MA) to remove cellular aggregates.

### Pulmonary Function Tests

All pulmonary function studies were performed in control and DS1-treated dogs before irradiation and at 5–7, 13 and 26 weeks after irradiation. Measurements of lung volumes and carbon monoxide diffusion capacity were performed on the right and left lungs separately, as described in *Methods S1* and in Nash et al. [15].

### Chest CT Imaging

Inspiratory chest CT scans were performed before and at 5 and 13 weeks after lung irradiation, as described in *Methods S1*. CT images were processed using an open source software program, OsiriX Imaging Software (http://www.osirix-viewer.com). 3D-images of the lungs were created using Irfanview and Volocity softwares, and saved in Quicktime QTVR movie.

### Immune Histochemistry (IHC) of TTF-1+ Cells and its Quantification

The detailed IHC procedures were described in *Methods S1*. In brief, IHC was performed to detect TTF-1+ cells in lung tissue collected only at necropsy. Lung tissue sections were incubated with anti-TFF-1 antibodies (rabbit monoclonal, Epitomics, Burlingame, CA) or a concentration-matched control rabbit IgG (negative control) and visualized using secondary antibodies (Mach2 rabbit HRP) with DAB and counter-stained with hematoxylin. Cells were imaged using a Nikon E800 microscope (Nikon, Tokyo, Japan) fitted with a 40×/1.30 Plan Fluor or a 100×/1.30 Plan Fluor objective lens. Bright contrast images were acquired on a Photonomics Coolsnap cf camera (Roper Scientific, Tucson, AZ).

The number of TTF-1+ cells in alveolar regions was quantified using HistoQuest software from Tissuegnostics (Vienna, Austria) as described in Figure S2. The quantification and analysis were performed by individuals blinded to the experimental conditions.

### Microscopy

Phase-contrast images of the cells were captured using an inverted phase/fluorescence microscope with a direct camera attachment (Diaphot-TMD: Nikon, Melville, NY).

### Semi-quantitative Reverse Transcriptase-polymerase Chain Reaction (RT-PCR)

RNA transcript levels for endothelial cell markers expressed by DS1 and ELC were quantified using semi-quantitative RT-PCR as described in *Methods S1*. Primers for dog transcripts are listed in Table S2.

### Endothelial-like Progenitor Cells (ELC) Colony Assay

Peripheral blood mononuclear cells (PBMCs) were isolated over a Ficoll-Hypaque step gradient (1.074 g/mL) and washed 3 times in HBSS. PBMCs (10 million cells) were plated in a T75 flask and cultured in supplemented RPMI 1640 medium containing 10% FCS, L-glutamine (0.4 mg/mL), sodium pyruvate (1 mmol/L), penicillin (100 U/mL) and streptomycin sulfate (100 pg/mL) for 1 week without changing the media. Non-adherent cells were discarded, and the adherent cells were cultured for additional 3–4 weeks. Conditioned media was gently replaced with fresh media weekly. Colonies of ELC larger than 2 mm in diameter were scored.

### Variable Number Tandem Repeat Analysis (VNTR)

VNTR analysis was conducted by the Genotype Tracking Laboratory of the CCEH (DK056465) at the FHCRC as previously described [16], [17].

### Statistics

Comparisons of lung function within groups of dogs were evaluated by Repeated Measures ANOVA (Fisher’s PLSD). Mean and standard deviations (SDs) were determined, and statistically significant differences were identified by paired and unpaired Student *t* test (p<0.05).

## Results

### Irradiation-induced Lung Injury and DS1 Cell Infusion

**Figure 1:**
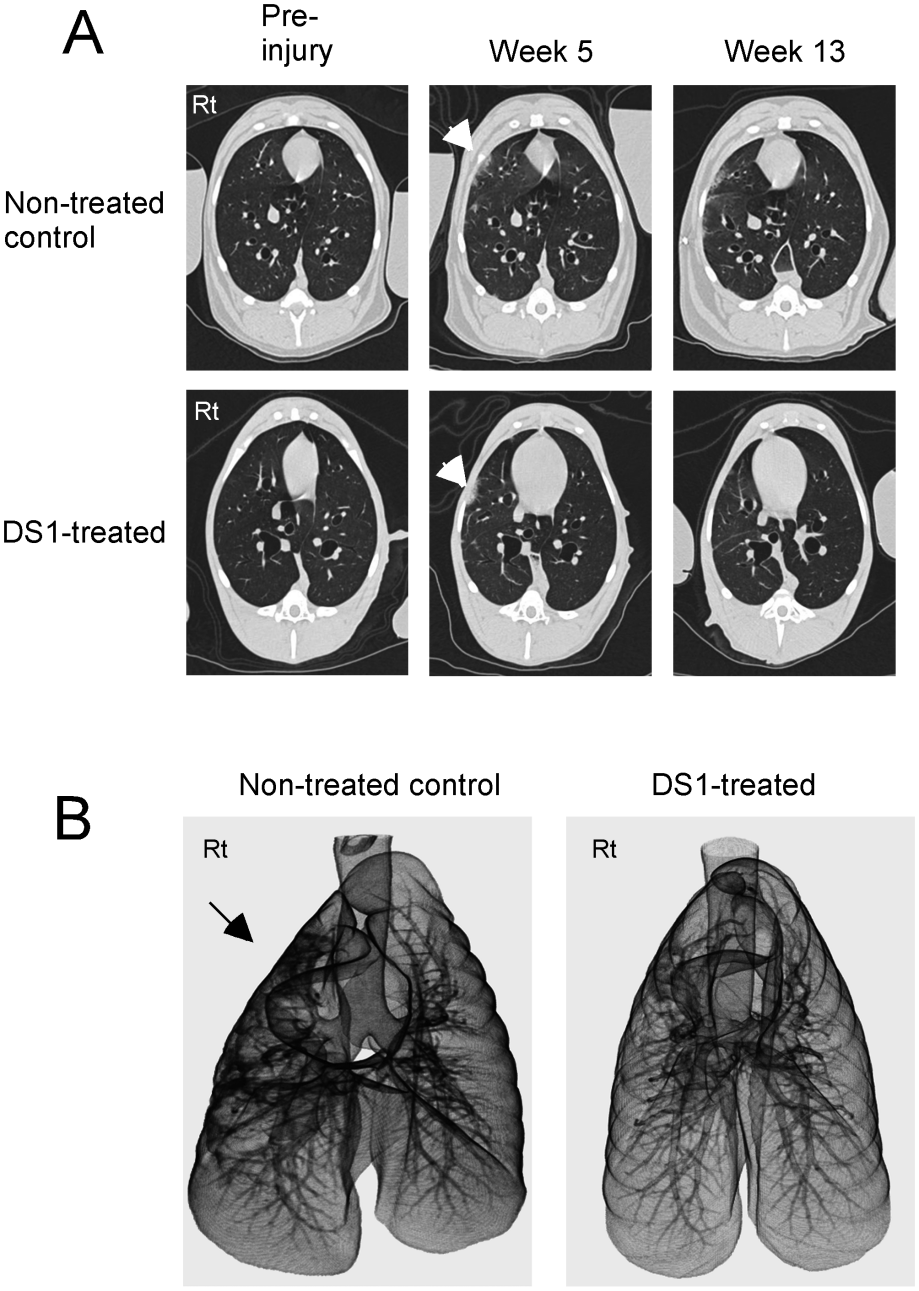
High resolution CT scans of the chest at baseline and then at the indicated time points after 15 Gy right lung irradiation and DS1 cell infusion. Panel A: At baseline, no abnormalities were detected in the lung parenchyma of either dog (Pre-injury). Five weeks after irradiation focal infiltrates were detected in the right lung of both dogs (indicated by the white arrow heads). The non-treated control dog had extensive diffuse interstitial changes in the irradiated right lung and loss of right lung volume at Week 13 (the top right image of panel A). In contrast, nearly complete resolution of the right lung infiltrates was seen in the DS1-treated dog at Week 13 (the bottom right image of panel A). Panel B: CT scans of the whole lung from the DS1-treated and control dog at week 13 were reconstructed in 3D-images. An arrow indicates the right lung of the control dog with extensive damage. These data indicate reversal of radiation-induced lung injury after DS1 administration.

Dogs in the DS1-treated group received a single infusion of DS1 cells 5 weeks after irradiation of the right lung. This time point was selected because previous pilot studies determined that focal infiltrates, indicative of radiation damage, could be detected by CT scan 5 weeks following irradiation. Figure 1 shows CT scan data of a control and DS1-treated dog. Focal infiltrates were detected in the right lung of both dogs 5 weeks after irradiation. By Week 13 the infiltrates were more extensive in the control dog and largely resolved in the DS1-treated dog.

Pulmonary function tests (PFTs) were performed on 10 dogs before and after 15 Gy right lung irradiation. The 5 control dogs that did not receive DS1 cells had a steady decrease in right lung function as measured by diffusion capacity (% DLCO-Hct), inspiratory capacity (IC), functional residual capacity (FRC), expiratory reserve volume (ERV) and total lung capacity (TLC), up to 13 weeks post irradiation, at which point values reached a plateau of 50–70% of the pre-irradiation baseline levels. All five dogs in the treated group were given a single infusion of DS1 cells at 5 weeks after irradiation. Although there was some variability, dogs receiving DS1 cells had significant improvement in right lung % DLCO-Hct values at Week 13 compared to the control dogs (p = 0.003, Table 1). Likewise, IC, ERV, RV and FRC were significantly improved in DS1 treated dogs at Week 13 (Table 1). Non-irradiated left lungs did not show any changes in lung function between the two groups at any time point.

**Table 1:** Pulmonary function tests (PFTs) of untreated and DS1 treated dogs at Week 13 post right lung irradiation.

	No DS1 infusion			+ DS1 infusion			*p* value		
	Right	Left	Total	Right	Left	Total	Right	Left	Total
**Pulmonary diffusion capacity for CO**									
% DLCO-Hct	54±9	115±15	70+10	85±14	125±35	99+10	0.003	*N.S.*	0.002
**Inspiratory**									
% IC	65±7	107±11	78±6	76±9	137±32	96±9	*0.05*	*N.S.*	*0.006*
**Expiratory**									
% ERV	64±16	130±22	81±8	94±17	140±25	111±14	*0.02*	*N.S.*	*0.004*
**Inspiratory+Expiratory**									
% TLC	68±6	114±19	82±6	82±9	150±33	106±12	*0.02*	*N.S.*	*0.004*
**Residual**									
% RV	67+21	109+28	79+21	141+44	128+34	137+29	*0.01*	*N.S.*	*0.007*
**Residual+Expiratory**									
% FRC	72±10	123±31	89±10	91±9	169±47	119±19	*0.01*	*N.S.*	*0.01*

Values are shown as means and standard deviations (SDs) at Week 13 (n = 5 for both groups).

*P values* were calculated between the control (No DS1 infusion) and the treated (+DS1 infusion) groups.

DLCO-Hct: Single breath diffusing capacity-hematocrit.

IC: Inspiratory capacity.

ERV: Expiratory reserve volume.

TLC: Total lung capacity.

RV: Residual volume directly obtained by neon dilution during the DLCO measurements.

FRC: Functional residual capacity.

Percentage (%) of PFTs was based on the pre-irradiation values.

N.S.: not significant.

PFT values obtained at 26 weeks, just prior to necropsy are summarized in Table 2. Although the level of lung function in the DS1 treated group decreased compared to Week 13, the DLCO-Hct, TLC and FRC remained significantly higher than those of the untreated dogs, and two of the five dogs sustained 100% recovery of pulmonary function.

**Table 2:** Pulmonary function tests (PFTs) of untreated and DS1 treated dogs at Week 26 post right lung irradiation.

	No DS1 infusion			+ DS1 infusion			*p* value		
	Right	Left	Total	Right	Left	Total	Right	Left	Total
**Pulmonary diffusion capacity for CO**									
% DLCO-Hct	54±14	113±24	70±17	75±14	124±36	92±21	0.05	*N.S.*	*N.S.*
**Inspiratory**									
% IC	61±10	118±9	79±8	66±12	145±40	92±18	*N.S.*	*N.S.*	*N.S.*
**Expiratory**									
% ERV	73±15	132±31	88±12	91±18	164±75	115±32	*N.S.*	*N.S.*	*N.S.*
**Inspiratory+Expiratory**									
% TLC	63±8	114±8	79±4	80±13	161±55	108±26	*0.03*	*N.S.*	*0.05*
**Residual**									
% RV	69±29	110±45	81±33	105±37	132±44	116±36	*N.S.*	*N.S.*	*N.S.*
**Residual+Expiratory**									
% FRC	66±10	111±13	80±5	95±21	185±76	126±39	*0.02*	*N.S.*	*0.03*

Values are shown as means and SDs at Week 26 (n = 5 for both groups).

DLCO-Hct: Single breath diffusion capacity-hematocrit.

IC: Inspiratory capacity.

ERV: Expiratory reserve volume.

TLC: Total lung capacity.

RV: Residual volume directly obtained by neon dilution during the DLCO measurements.

FRC: Functional residual capacity.

Percentage (%) of PFTs was based on the pre-irradiation values.

N.S.: not significant.

### Immune Histochemistry (IHC) of TTF-1 in Lung Tissue

**Figure 2:**
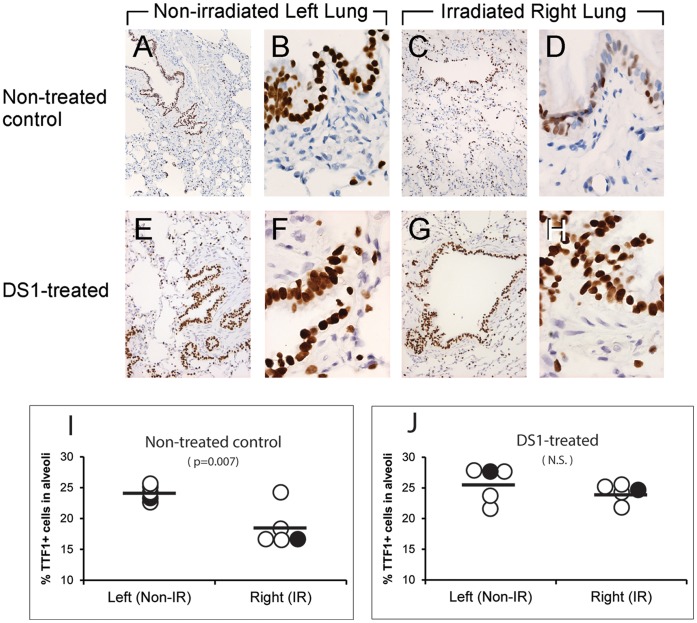
Immune histochemistry and quantitation of TTF-1+ cells in bronchioles of a non-treated control dog (Panels A to D) and a DS1-treated dog (Panels E and H) at necropsy (week 26). Two columns on the left are from non-irradiated left lungs; two columns on the right are from irradiated right lungs. TTF-1 was detected brown with HRP-conjugated antibodies and DAB as a chromogen. Nuclei were counterstained with hematoxylin (blue). Original objective X20 for Panels A, C, E and G; X100 for Panels B, D, F, and H. **Panels I and J:** The number of TTF-1+ cells in alveolar regions was quantified using HistoQuest software as described in Figure S2. The percentage of TTF-1+ cells in left and right lungs of the non-treated control dogs (n = 5) and DS1-treated dogs (n = 5) at necropsy (Week 26) are shown in Panels I and J, respectively. IR = irradiation. Horizontal bars indicate mean values. The solid circles indicate the values for the representative images shown in A and C for panel I, and E and G for panel J.

Thyroid transcription factor 1/NK2 homeobox 1 (TTF-1/NKX2-1) is a protein that regulates transcription of genes specific to the lung, thyroid and diencephalon. TTF-1 can be detected in type II pneumocytes in alveoli and in Clara cells/non-ciliated cells in bronchioles. Lungs from the non-treated control and DS1-treated dogs were obtained at the end of study (Week 26) and stained for TTF-1 by IHC (Figure 2). Figure S1 shows the specificity of the immunostaining using an isotype control and an irrelevant antibody, anti- Von Willebrand factor (vWF). In Figure 2, multilayered columnar cells in bronchioles of the non-irradiated left lung are positive for TTF-1 in all dogs. However, the radiation injured right lung of the control dogs had reduced numbers of TTF-1+ cells (Figure 2D), whereas the dogs that received and responded to DS1 infusion had intact TTF-1+ cells in the bronchioles (Figure 2H). The number of TTF-1+ cells in alveolar regions was digitally quantified using HistoQuest software by individuals blinded to the experimental conditions as described in Methods and Figure S2. The control dogs had a significant reduction in TTF-1+ cells in alveoli of the irradiated lung compared to the non-irradiated lung (18.6±3.3% and 24.2±1.2% TTF-1+ cells respectively; p = 0.007 by paired Student *t* test) (Figure 2I). In contrast, there was no difference in TTF-1+ cells between the irradiated and non-irradiated lungs in the four dogs that received and responded to DS1 infusion (23.8±1.1% and 25.1±3.1% TTF-1+ cells, respectively; not significant) (Figure 2J). Whether this difference represents protection from cell loss or accelerated regeneration remains to be determined.

### Detection of Endothelial-like Progenitor Cells (ELC) in Blood of the DS1 Treated Dogs

**Figure 3:**
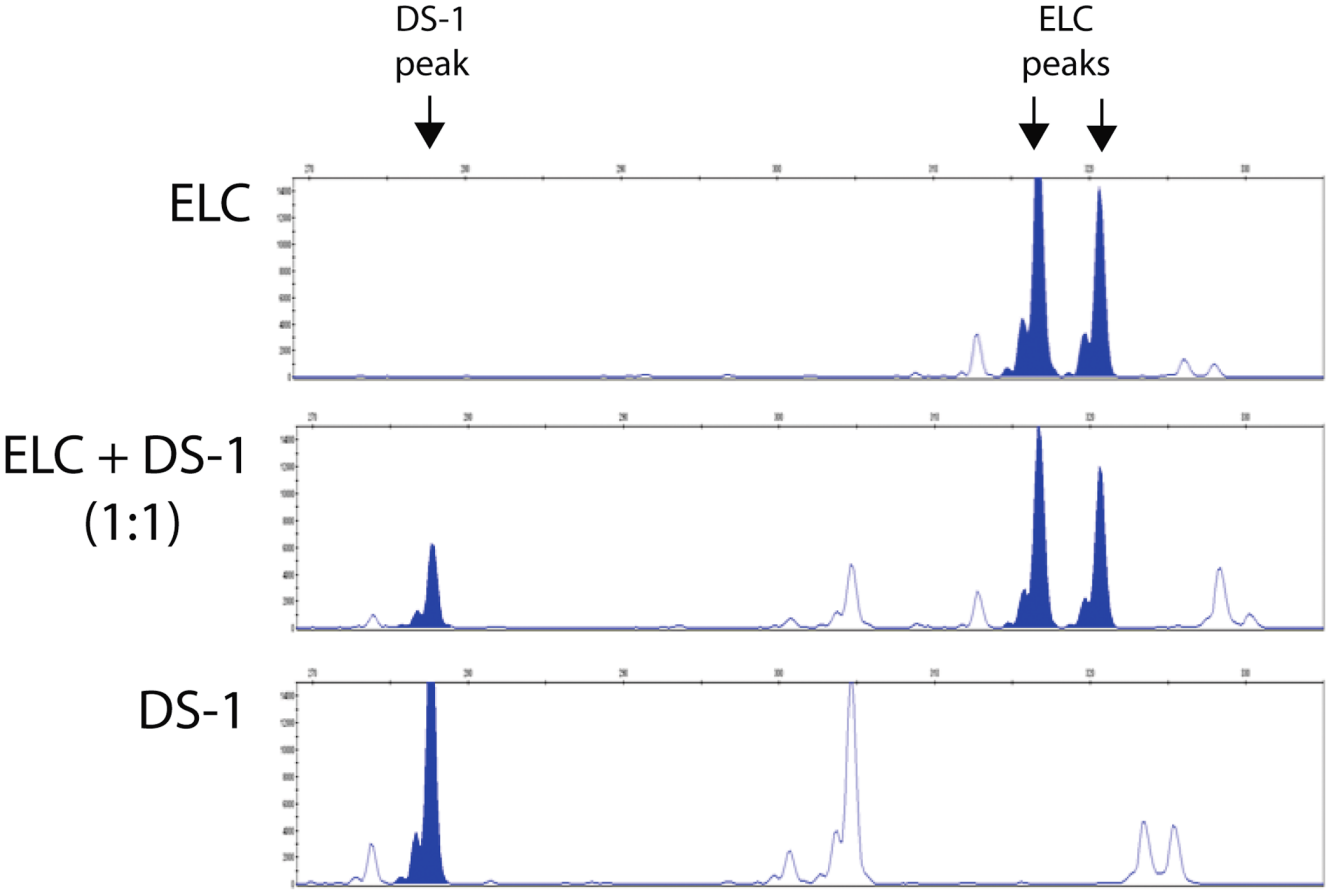
Variable number tandem repeat (VNTR) assay. Genomic DNA of ELC and DS1 cells were isolated and amplified by PCR for VNTR assay (top and bottom panels, respectively). The middle panel shows the VNTR of pre-mixed genomic DNA from both cells at 1∶1 ratio. Peaks of the unique tandem repeats with good separation were shown by filled peaks. The pattern of VNTR from ELC and DS1 cells are distinct, indicating that ELC cells are host in origin but not DS1 cells.

**Figure 4:**
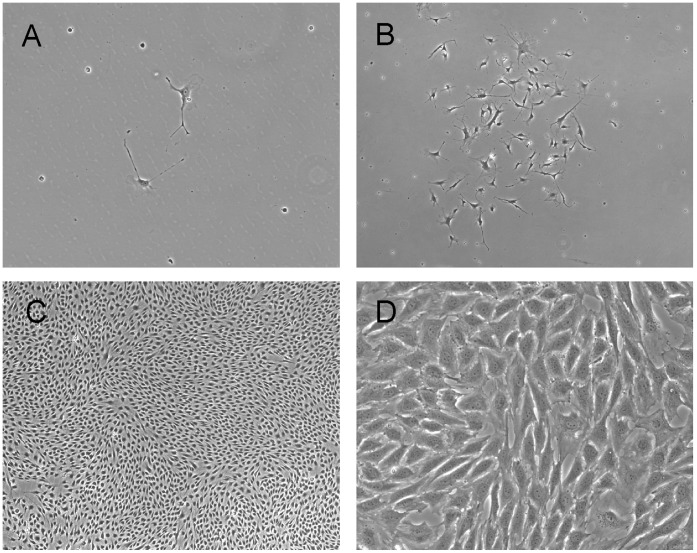
Phase-contrast images of ELC colonies. PBMC from a DS1-treated dog were isolated 4 days post DS-1 cell infusion, and cultured *in vitro*. Two adherent cells appeared 4 days after the start of culture as shown in Panel A. Panel B shows the expansion of the 2-cell colony to a small ELC colony 10 days later. Panel C shows an image of the center of the colony 15 days after Panel B. Panel D shows a higher magnification of Panel C. Original objective: 10X for Panel A, 4X for Panels B and C, and 20X for Panel D.

**Figure 5:**
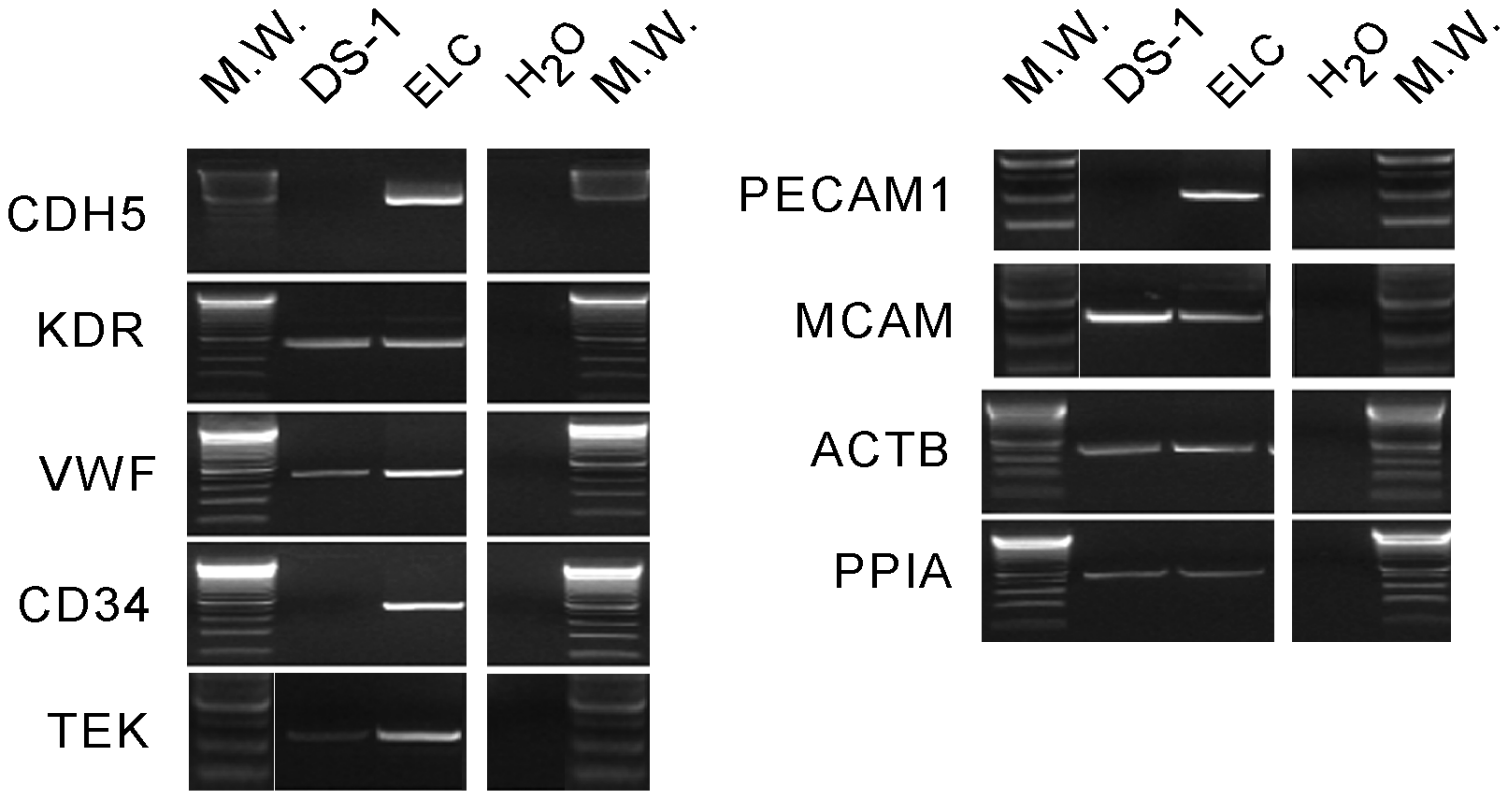
Reverse transcriptase-polymerase chain reaction (RT-PCR) of ELC. Expression levels of endothelial cell and house keeping genes were determined in ELC and DS-1 cells by semi-quantitative RT-PCR. cDNA of the cells was normalized using beta actin and cyclophilin A. PCR primers for the dog gene transcripts were used for CDH5 (cadherin 5, VE-cadherin), KDR (kinase insert domain receptor, FLK1, VEGFR2), VWF (von Willebrend factor), CD34, TEK (Tie-2), PECAM1 (CD31), MCAM (CD146), ACTB (beta actin), and PPIA (cyclophilin A, peptidylprolyl isomerase A). H_2_O represents a negative control (without cDNA).

ELC colony assay was performed in DS1-treated dogs (n = 5) at pre-DS1 infusion (before and after irradiation) and at multiple time points after DS1 infusion (3–6 hours, and 1, 2, 4, 7, 14, 21, 28 and >35 days post-DS1). Peripheral blood mononuclear cells (PBMC) were harvested from DS1-treated dogs and cultured for 30 days under conditions that support the growth of the ELC colonies. ELC colony forming units were detected in peripheral blood 1 to 7 days after DS1 cell infusion, but not pre-DS1 infusion or more than 2 weeks after infusion. PCR-based analysis of the variable number tandem repeats (VNTR) in satellite DNA showed that these ELC colonies were not derived from DS1 cells (Figure 3). ELC colonies grew slowly and were first detected between 7–10 days after the start of culture. After 30 days in culture they reached a median size of 8 mm in diameter ranging from 5–13 mm (Figure 4 and Figure S3). Given that reliable antibodies for canine endothelial determinants are not available, RT-PCR was used to characterize the ELC. RT-PCR shows that ELC colonies contained standard transcripts seen in endothelial cells including CDH5/VE-cadherin, CD34, TEK/TIE2, and PECAM1/CD31 (Figure 5).

## Discussion

Our canine model of radiation-induced lung injury reproduces with high fidelity the radiographic, physiological, and immunological characteristics of radiation-induced lung injury observed in patients treated with thoracic radiation. Importantly, the severity and time-dependent progression of lung damage in this model are not affected by concomitant injury to the hematopoietic compartment or by supportive measures used to restore the bone marrow. Therefore, this model serves as a relevant platform for the development of strategies to mitigate radiation-induced damage to the lung.

In this study, we showed that a single i.v. infusion of DS1 marrow fibroblasts, given 5 weeks after irradiation, prevented or delayed the progression of radiation-induced lung injury. At Week 13 after irradiation, 4 of 5 DS1-treated dogs showed improved pulmonary function in their irradiated right lungs with mean values of DLCO-Hct, IC, ERV and TLC, within the normal range and significantly higher than the right lungs of control dogs (n = 5 for each group, p<0.0476, Fisher’s Exact). Non-irradiated left lungs did not show any differences in PFTs between the treated and control dogs. DS1-treated dogs also had less pneumonitis, detected by CT scan imaging, and significantly more TTF1+ cells detected by IHC, in the bronchioles and alveoli compared to control dogs. Since epithelial repair is thought to be critical for preventing fibrosis, we hypothesize that the re-epithelization suggested by increased TTF1+ cells may contribute to a reduction in fibrosis and improved lung function.

DS1 is a cloned cell line derived from a primary culture of bone marrow stromal cells. DS1 cells represent a single cell type present in an otherwise heterogeneous population of cells referred to as mesenchymal stromal cells (MSC). Mechanistic studies using primary MSC to treat lung injury have been performed in mouse models [18]–[23]. The majority of these studies suggest that rodent MSC mitigate lung injury by stimulating an anti-inflammatory response. Only limited information exists regarding the effect of MSCs in human lung injury. Marrow-derived MSCs have been shown to enhance alveolar fluid clearance in an *ex vivo* perfused human lung model, but whether these results can be applied to injured human lungs *in vivo* is uncertain [24]. Here we used a canine model of lung injury since our experience with other disease models has shown that outcomes in canines accurately predicts outcomes in patients [25].

The DS1 cell infusion also induced transient mobilization of host-derived endothelial-like progenitor cells (ELC) into the circulation. In a previously published report a rapid increase of endothelial progenitor cells in the circulation was associated with acute stress injuries of the vascular endothelium such as acute lung injury [26]. Additional reports indicate that following acute lung injury, patients with higher numbers of circulating endothelial cells have significantly higher survival rates after controlling for age, gender, and severity of illness, suggesting that robust mobilization of endothelial progenitor cells may contribute to the repair of the injured pulmonary endothelium [27]–[29]. Our data from the untreated control dogs showed that these endogenous repair/defense mechanisms were not sufficient to restore lung function following 15 Gy irradiation. However, since ELC mobilization increased following DS1 infusion, and this in turn was associated with recovery of lung function, we hypothesize that DS1-stimulated ELC mobilization boosted these endogenous repair mechanisms.

The data presented in this study provide a characterization of the ELC that appear in the blood after the infusion of DS1. How these ELC relate to rodent and human endothelial progenitor cells previously reported to support endothelial regeneration is unclear. The primary cultures of ELC clearly include endothelial cells expressing VE-cadherin, CD34, KDR, vWF, TEK/TIE2, and PECAM1, which are commonly used markers to identify endothelial progenitors [30]. Using variable numbers of tandem repeats (VNTR), a polymorphic feature of satellite DNA that can distinguish between dogs, the ELCs are clearly of recipient origin. ELC can also be distinguished from DS1 by gene expression data. Given that the ELCs are similar to endothelial progenitors, their mobilization may rely on the same pathways. Potent mobilization stimuli include hypoxia through hypoxia-inducible factor-1 (HIF-1)-induced expression of stromal cell derived factor-1 (SDF-1/CXCL12), nitric oxide, VEGF, EPO, and G-CSF [27], [31]. Gene expression profiles of DS1 cells using the Affymetrix microarray platform show that DS1 cells express transcripts of pro-angiogenic factors such as VEGF, PDGF and IGFBP and pro-inflammatory genes such as IL-8 and MCP1 (http://webapps.fhcrc.org/labs/graf/grantdata.html) [14]. All or a combination of these factors may promote angiogenesis and ELC mobilization. In mice, endothelial progenitor cells are thought to arise from both the bone marrow and vascular compartments [32]. Single hematopoietic stem cell transplantation was reported to generate vascular endothelium in mice [33]; yet progenitors [34], [35] that participate in postnatal neovascularization were also reported to originate from within the vascular wall [36]. The current study does not address the source of the circulating ELC or whether the circulating ELC engraft in the right lung: the ELC are autologous cells and cannot be distinguished from other resident cells. Dogs that are hematopoietic chimeras could be used to determine whether the ELC are marrow-derived [37], [38]; however, earlier studies with chimeric dogs preceded some technical advances that could make such studies more unequivocal.

Although DS1-treated dogs showed improved pulmonary function at Week 13, only two dogs sustained this level of function until the end of study. However, it should be noted that only a single DS1 cell infusion given at one time point was used in this study; therefore, it is possible that a more optimal injection schedule could result in a more uniform and permanent outcome. Additionally, we also hypothesize that the in vivo canine model will accurately predict outcomes in human patients. This hypothesis is based on a large body of canine studies that accurately predicted human transplantation outcomes, as well as the outcomes from treatments of transplant complications [25], [39], [40]. However, studies to identify a human fibroblast equivalent of DS1, the activities responsible for this effect, and an optimal treatment schedule are needed before translating this treatment to human patients.

## Supporting Information

Figure S1 Immune histochemistry of TTF1 and vWF in canine lung.
(DOCX)

Figure S2 Quantification of TTF-1 positive cells in immune histochemistry.
(DOCX)

Figure S3 Kinetics of ELC colony assay and growth of the ELC colonies.
(DOCX)

Figure S4 Schematic diagram of experimental design for lung irradiation and DS1 cell infusion.
(DOCX)

Table S1 Demographics of the dogs.
(DOCX)

Table S2 List of the primers.
(DOCX)

Methods S1 (DOCX)
