# Supplementary material for: Late Infusion of Cloned Marrow Fibroblasts Stimulates Endogenous Recovery from Radiation-Induced Lung Injury
Source: PLoS One. 2013 Mar 8;8(3):e57179. doi: 10.1371/journal.pone.0057179 (PMC3592849; doi:10.1371/journal.pone.0057179)
Supplement: Figure S1 — Immune histochemistry of TTF1 and vWF in canine lung. (DOCX) [file pone.0057179.s001.docx]

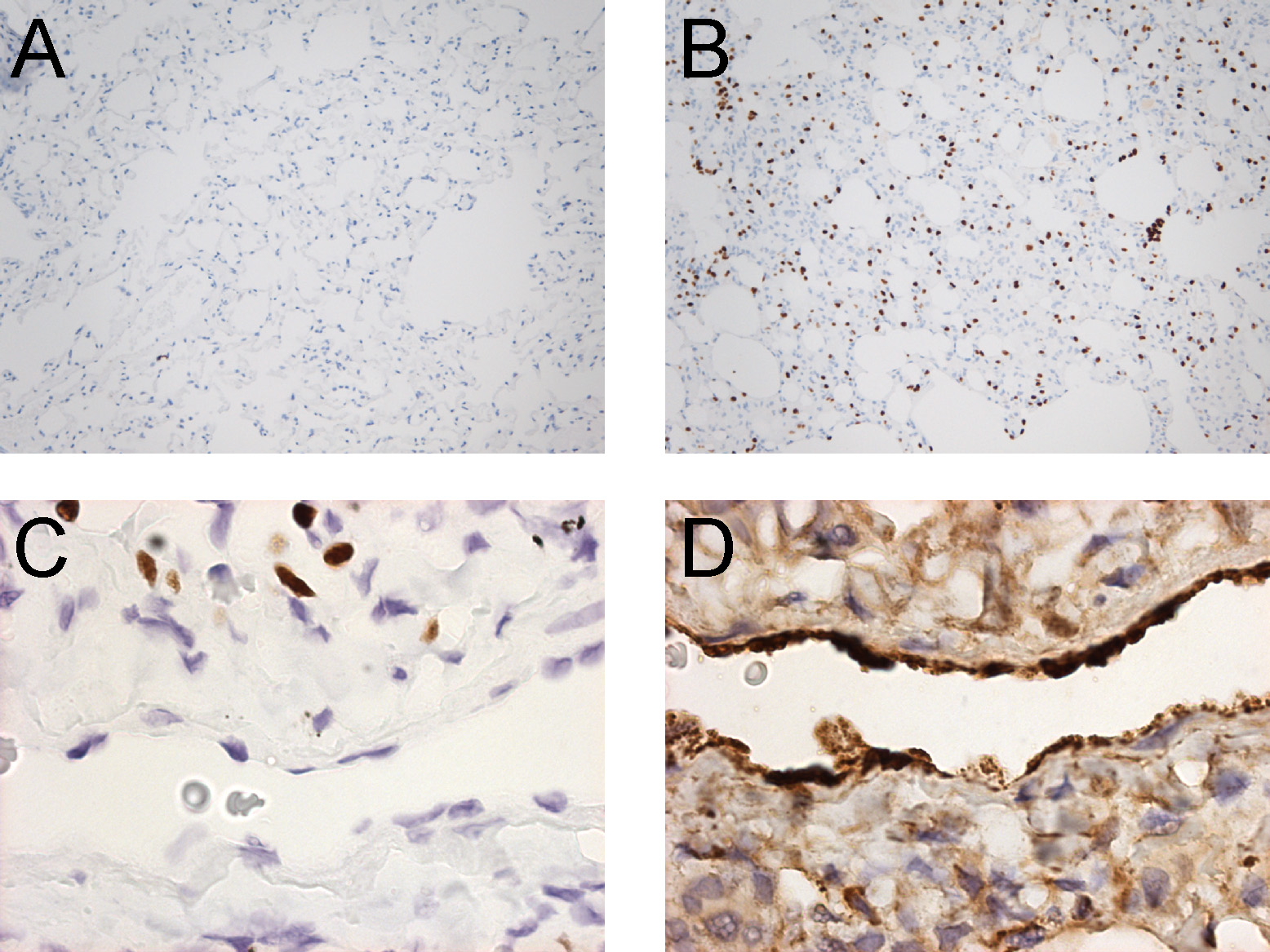


**Figure S1. Immune histochemistry of TTF1 and vWF in canine lung.** Panels A and B: Alveoli were incubated with concentration-matched rabbit IgG and anti-TTF1 antibodies, respectively. Bound antibodies were detected with HRP-conjugated secondary antibodies and DAB (brown staining). Nuclei were counter-stained with hematoxylin (blue). Original objective, X20. Panels C and D: Endothelial cells in lung capillary are negative for TTF1 but positive for vWF (Panels C and D, respectively). Original objective, X100.
