# Supplementary material for: Late Infusion of Cloned Marrow Fibroblasts Stimulates Endogenous Recovery from Radiation-Induced Lung Injury
Source: PLoS One. 2013 Mar 8;8(3):e57179. doi: 10.1371/journal.pone.0057179 (PMC3592849; doi:10.1371/journal.pone.0057179)
Supplement: Figure S2 — Quantification of TTF-1 positive cells in immune histochemistry. (DOCX) [file pone.0057179.s002.docx]

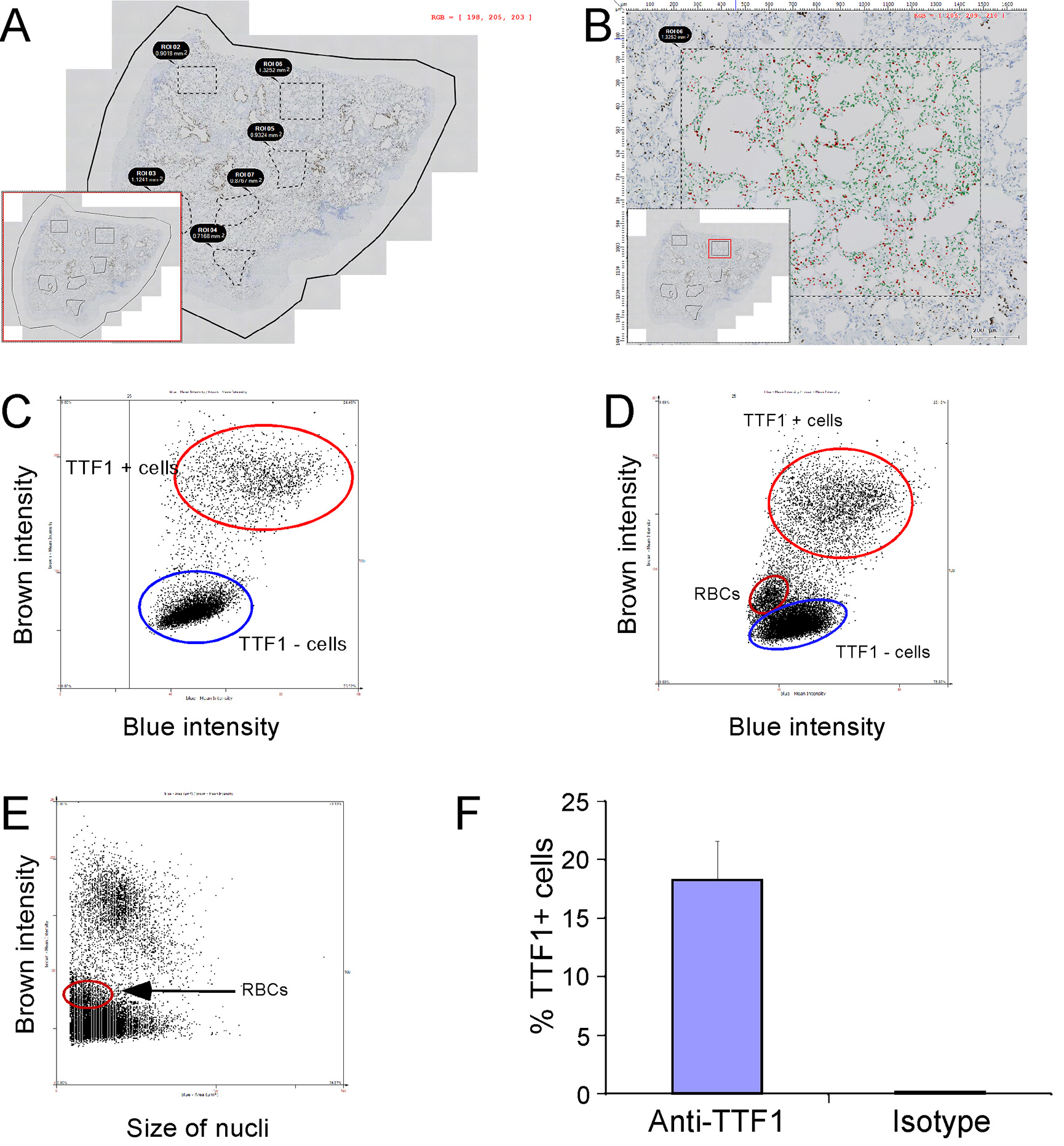


**Figure S2. Quantification of TTF-1 positive cells in immune histochemistry.** In order to quantify TTF-1+ cells in alveoli of DS1 treated and untreated dogs, HistoQuest software from Tissuegnostics (Vienna, Austria) was used. Panel A: Immune histochemistry of TTF-1 was conducted where brown and blue nuclei were TTF-1 positive and negative cells, respectively. Six areas on the section of alveoli were chosen blindly. Panel B: One of the six areas was selected (the red square in the insert), and color and size segmentations were applied to separate TTF-1+ and TTF-1 negative nuclei and to identify single cells. TTF-1 positive and negative cells were pseudo-colored in red and green, respectively. Panels C and D: Intensity of brown (TTF-1+ cells) and blue (TTF-1- cells) staining from non-irradiated control and irradiated lungs, respectively. Red blood cells (RBCs) were identified in the irradiated lung in Panel D. Size of RBCs was smaller than alveolar cells as expected (Panel E). Panel F: Immune histochemistry using anti-TTF1 antibodies and concentration-matched rabbit IgG (Isotype) was conducted. % of TTF1+ cells were calculated as a ratio of brown cell number over brown+blue cell number. The values are represented as an average of 3 sections (>6 areas per section).
