# Supplementary material for: Late Infusion of Cloned Marrow Fibroblasts Stimulates Endogenous Recovery from Radiation-Induced Lung Injury
Source: PLoS One. 2013 Mar 8;8(3):e57179. doi: 10.1371/journal.pone.0057179 (PMC3592849; doi:10.1371/journal.pone.0057179)
Supplement: Figure S3 — Kinetics of ELC colony assay and growth of the ELC colonies. (DOCX) [file pone.0057179.s003.docx]

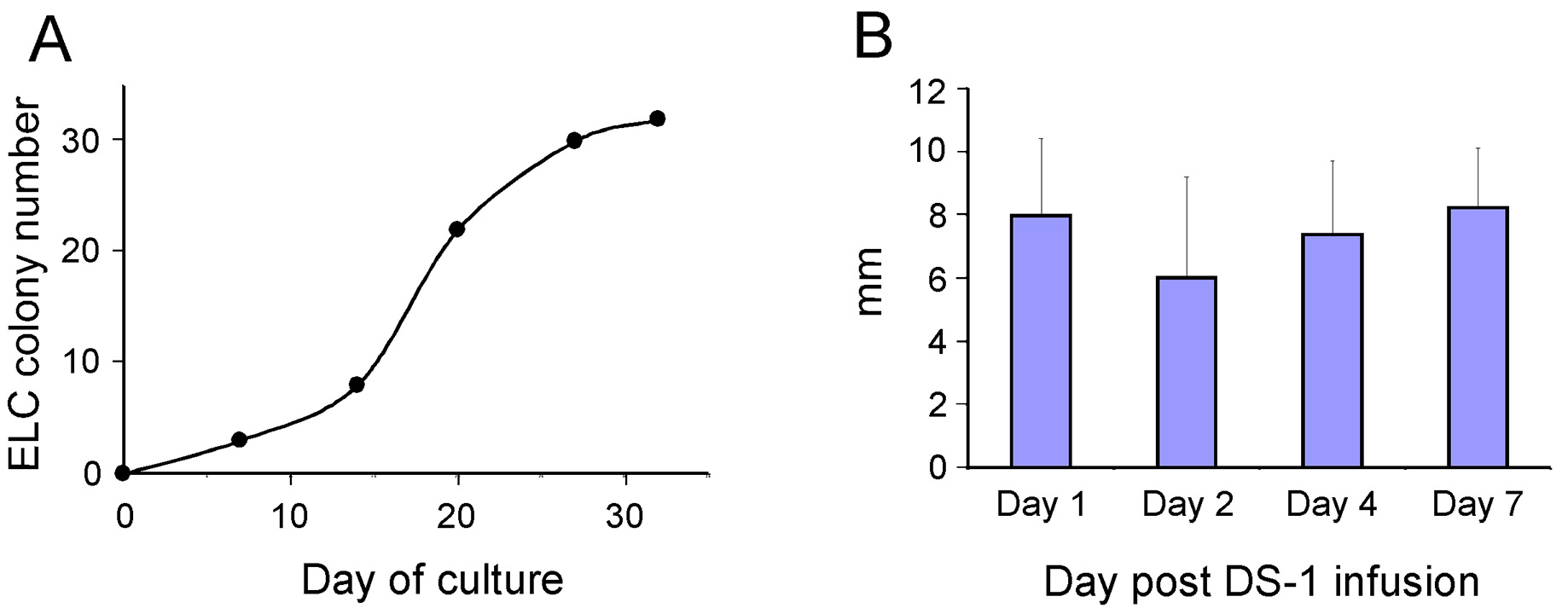


**Figure S3. Kinetics of ELC colony assay and growth of the colonies. Panel A:** PBMC (10×10^6^) from one of the DS1 dogs (H332) were harvested one day post DS-1 infusion, and cultured for 31 day as shown in X-axis. Number of ELC colonies bigger than 2 mm diameter was scored. **Panel B:** PBMC of the same dog (H332) were harvested 1 to 7 days post DS-1 infusion, and cultured for 30-31 days. Size of the colonies was measured (mm in diameter), and means+/-SDs were calculated (n=32, 2, 4, and 30 for Day 1, 2, 4, and 7, respectively).
