# Supplementary figures and images for: Late Infusion of Cloned Marrow Fibroblasts Stimulates Endogenous Recovery from Radiation-Induced Lung Injury
Source: PLoS One. 2013 Mar 8;8(3):e57179. doi: 10.1371/journal.pone.0057179 (PMC3592849; doi:10.1371/journal.pone.0057179)

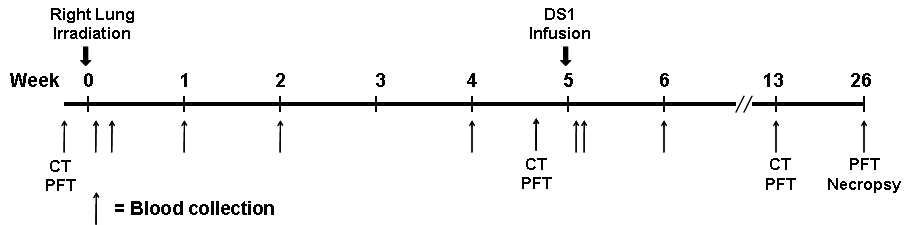


**Figure S4. Schematic diagram of experimental design for lung irradiation and DS1 cell infusion.**

Supplement: Figure S4 — Schematic diagram of experimental design for lung irradiation and DS1 cell infusion. (DOCX) [file pone.0057179.s004.docx]
