# Supplementary material for: Late Infusion of Cloned Marrow Fibroblasts Stimulates Endogenous Recovery from Radiation-Induced Lung Injury
Source: PLoS One. 2013 Mar 8;8(3):e57179. doi: 10.1371/journal.pone.0057179 (PMC3592849; doi:10.1371/journal.pone.0057179)
Supplement: Table S1 — Demographics of the dogs. (DOCX) [file pone.0057179.s005.docx]

**Table S1. Demographics of the dogs**

|  |  |  |  |  |
| --- | --- | --- | --- | --- |
| Recipient | Sex | Age at irradiation (Mo) | Weight at irradiation (Kg) | DRB allele |
|  |  |  |  |  |
| Dogs with DS-1 infusion | | |  |  |
| H303 | F | 15.9 | 13.0 | DRB1*006:01, DRB1*015:01 |
| H332 | M | 10.5 | 11.9 | DRB1*001:02, DRB1*006:01 |
| H336 | M | 15.5 | 11.0 | DRB1*006:01, DRB1*015:01 |
| H447 | F | 6.7 | 12.4 | DRB1*001:02, DRB1*009:01 |
| H450 | M | 8.6 | 18.5 | DRB1*006:01, DRB1*011:01 |
|  |  |  |  |  |
| Control dogs | |  |  |  |
| H065 | M | 23.7 | 15.7 | DRB1*008:01, DRB1*017:01 |
| H136 | M | 16.8 | 11.8 | DRB1*019:01, DRB1*024:01 |
| H181 | F | 11.9 | 10.4 | DRB1*009:01, DRB1*015:01 |
| H202 | F | 8.6 | 13.8 | DRB1*002:01, DRB1*002:01 |
| H233 | M | 8.9 | 10.5 | DRB1*006:01, DRB1*015:01 |
|  |  |  |  |  |
| Dog of DS1 origin | |  |  |  |
| G060 | F | (not irradiated) | (not irradiated) | DRB1*001:02, DRB1*006:01 |
|  |  |  |  |  |
