# Supplementary material for: Late Infusion of Cloned Marrow Fibroblasts Stimulates Endogenous Recovery from Radiation-Induced Lung Injury
Source: PLoS One. 2013 Mar 8;8(3):e57179. doi: 10.1371/journal.pone.0057179 (PMC3592849; doi:10.1371/journal.pone.0057179)
Supplement: Table S2 — List of the primers. (DOCX) [file pone.0057179.s006.docx]

| **Supplemental Table S2.** List of dog primers | |  |
| --- | --- | --- |
|  | 5' primer | 3' primer |
| Canine CD34 | CAGAAACCGTGATTACTCCTACCAC | AGCTCTAGGCAGATACCTTGGTTC |
| Canine CDH5/CD144/VE-cadherin | CACAGCCACAGTACTGGTCAACC | TGCGGATGGAATATCCAATGCTCC |
| Canine KDR | GAACTGAAGACAGGCTACTTGTCC | CTCTGACTACTGGTGATGCTGTCC |
| Canine vWF | GTCAGATTCAACCATCTTG GCCAC | GGGATGGTGGACATGACATAGCAC |
| Canine TEK/TIE2 | CTTTAAGATACAGCCTTTCCCATCC | CTGGTTCATTAAGGCTTCAAAGTCC |
| Canine PECAM1/CD31 | CTACCAAATCACCTCGAATGAAACC | TTCTGTGTATTCCACATCCAACGTC |
| Canine MCAM/CD146 | GGAGTCTCAGGAAGTCACTGTCC | CTTCAGGTTGTGTAACTGGAGCAC |
| Canine ACTB | GATGACGATATCGCTGCGCTTGTG | CATCACGATGCCAGTGGTGCGG |
| Canine PPIA/CYPA | CCGTGTTCTTTGACATCGCCGTG | GTTCAGATAAAACAGGAGTTAAGATTC |
