# Supplementary material for: Late Infusion of Cloned Marrow Fibroblasts Stimulates Endogenous Recovery from Radiation-Induced Lung Injury
Source: PLoS One. 2013 Mar 8;8(3):e57179. doi: 10.1371/journal.pone.0057179 (PMC3592849; doi:10.1371/journal.pone.0057179)
Supplement: Methods S1 — (DOCX) [file pone.0057179.s007.docx]

**Supporting Information for**

**Infusion of Cloned Marrow Fibroblasts Mitigates Radiation-Induced Lung Injury**

**Supplemental Methods**

Details of the following protocols were described by Madtes et al (invited revision, Radiation Research).

**Lung irradiation**

Lung irradiation was conducted by a third party clinical medical physics services (Northwest Medical Physics Center, Lynnwood, WA) that was blinded to the experimental condition. For right lung irradiation, dogs were anesthetized with a continuous intravenous infusion of propofol titrated to provide a surgical plane of anesthesia. The anesthetized animal was then intubated and placed in the left lateral position onto a lung irradiation platform so that the right forelimb was secured away from the radiation port using a soft limb restraint. The animal was positioned by a laser guide in front of the linear accelerator source so that the center of the thorax was 100 cm from the radiation source. The irradiation platform was elevated as needed to place the right lung within the radiation port, and chest x-rays using the linear accelerator were obtained to confirm the correct positioning. The irradiated areas included the right lung, the right hemi-diaphragm and the superior portions of the right side of the liver, and an approximately 5 mm strip of thymus adjacent to the apex of the lung. A lead shield was placed on the linear accelerator port to attenuate the dose of radiation to the heart with the dose point placed 1 cm above the heart block shadows. Once the correct position of the right lung within the radiation port was confirmed, irradiation of the right lung was performed with a dose of 2.5 Gy at 0.07 Gy per minute followed by a radiation boost of 5 Gy at 5 Gy/minute in the ventral to dorsal position measured as a mid-lung, center of field dose. The radiation dose delivered was confirmed using an external dosimeter placed within the radiation field on the dog’s chest, assuring equivalent radiation exposure to the right lung of each dog. The dog was then rotated 180 degrees so that the animal faced away from the linear accelerator source (still lying on the animal’s left side) positioned 100 cm from the radiation source. The position of the right lung within the radiation port was again confirmed by chest x-rays as described above. Once the correct position of the right lung within the radiation port was confirmed, irradiation of the right lung was performed with a dose of 2.5 Gy at 0.07 Gy/min followed by a radiation boost of 5 Gy at 5 Gy/minute in the dorsal to ventral position measured as a mid-lung, center of field dose. The total radiation dose to the right lung was 15 Gy (5 Gy at a dose rate of 0.07 Gy/min plus 10 Gy at 5 Gy/min). A calculated estimate of the radiation dose to the heart based on the lead shield as a percentage of prescribed right lung dose was 5% due to block transmission and another 5% from scatter. This dose of heart irradiation is not expected to have any negative effects.

**Pulmonary function tests**

For pulmonary function testing, each animal was anesthetized with a continuous intravenous infusion of propofol titrated to provide a surgical plane of anesthesia. A bifurcated endotracheal tube was placed to isolate the irradiated and non-irradiated lungs and isolation verified by checking for an airtight seal. Recipients were ventilated with a Harvard piston ventilator with a tidal volume of 12 ml/kg and a rate to produce an end tidal CO_2_ of 35-40 torr. The ventilator circuit was constructed to allow the total tidal volume to be distributed to the right and left lungs with equal airway pressures. Small one-way valves were used to minimize the dead space in the system. Dogs were maintained in the prone posture, and 15 cm H_2_O of PEEP was used regularly for 3-5 breaths to recruit lung volume and minimize atelectasis. Dogs were paralyzed with atracurium. Pulmonary function studies were performed on each lung in isolation. Measurements of lung volumes, carbon monoxide diffusion capacity, airway resistance and dynamic compliance were performed as previously described.[15]. The total lung capacity was determined by inflating the lungs to an airway of +25 cm H_2_O. The residual volume was determined by deflating the lungs to an airway pressure of -20 cm H_2_O. A modified form of the single-breath carbon monoxide test [41] was employed to measure DLCO simultaneously in both lungs. The dog was removed from the ventilator, and the lungs were deflated to residual volume (-20 cm H_2_O), then the individual lungs were simultaneously inflated to their pre-determined TLC volumes with gas containing 0.3% carbon monoxide and 0.5% neon in air. System dead space was withdrawn, then 150 mls of alveolar air was collected in separate syringes for analysis by gas chromatography (Medgraphics Profiler, St Paul, MN). The time of breath holding (t) was measured from ½ lung volume inflation to the time at which withdrawal of dead space gas was complete. DLCO was calculated from the equation of (VA × 60/(Pbar – 47) × t)) × (ln PACOi/PACOt)[42] and corrected for hemoglobin concentration. Alveolar volume (VA) was calculated from the inspired and expired neon fractions and the inflation volume.

**Canine chest CT scan**

The anesthetized animal was intubated and then placed in the supine position in a v-trough so that the ventral side of the animal was facing upward and the hind quarters were closest to the CT gantry. A Harvard piston ventilator (pressure limit 20 cm H_2_O) was used to ventilate the intubated dog with 30% O_2_ during the CT procedure. The dog’s airway pressure, end tidal CO_2_, O_2_sat, and temperature were monitored throughout the procedure. The dog was connected to the ventilator through one side of a 4-way valve. One of the three remaining ports of the 4-way valve was connected back to the ventilator and set to an end expiratory pressure (PEEP, 3 cm H_2_O) by a pressure-limiting valve. End expiratory pressure was raised to 10 cm H_2_O for 10-15 breaths to recruit lung volume and minimize atelectasis. The third port was connected to a static pressure source, while the remaining port was connected to a respiratory bag to enable continuous cycling of the ventilator when the circuit was closed to the endotracheal tube. To obtain a CT scan at end inspiration, the 4-way valve was rotated such that the dog’s lung was held at the set static pressure ~15-16 cm H_2_O and the ventilator ventilated the respiratory bag. The animal was permitted a rest period of at least 5 minutes between scans, during which time normal ventilation occurred. To obtain a CT scan at end expiration, the 4-way valve was rotated such that the dog’s lung was held at the set static pressure of PEEP (0 cm H_2_O) and the ventilator ventilated the respiratory bag. After completing the expiratory images, the CT images were visualized and assessed to assure adequate quality. If the CT images are judged to be of inadequate quality, then the image acquisition maneuvers were repeated.

**Immune histochemistry (IHC) of thyroid transcription factor-1 (TTF-1) positive cells**

The Experimental Histopathology Laboratory of the Shared Resources at the Fred Hutchinson Cancer Research Center (FHCRC) performed IHC to detect TTF-1+ cells in the lung biopsies. Lung tissue was immediately fixed in 10% neutral buffered formalin, then processed and embedded in paraffin. Sections (5 μm) were de-paraffinized and rehydrated in distilled water. Heat-induced epitope retrieval was then performed with a Citrate-based 0.05% Tween20 solution (pH 6.0, Dako) for 20 minutes in a steamer, followed by a 20-minute cool down. Slides were washed in Tris-based saline containing 0.05% Tween20 (TBS-T) and incubated with 3% hydrogen peroxide for 8 minutes to block endogenous peroxidase activity. The sections were then incubated in 50mM Tris-HCl, pH7.6, containing 0.15M NaCl, 0.25% (w/w) casein and 0.1% Tween 20 for 10 minutes to block non-specific protein binding. They were incubated with anti-TFF-1 antibodies (rabbit monoclonal, Epitomics) or a concentration matched control rabbit IgG as a negative control. Slides were washed with TBS-T, incubated with secondary antibodies (Mach2 rabbit HRP) for 30 minutes, washed again, and then incubated further in DAB for 4 minutes twice. Nuclei were counter-stained with hematoxylin before cover-slipped with Crystal mount.

**Immune histochemistry (IHC) of von Willebrand factor (vWF) positive endothelial cells**

The Experimental Histopathology Laboratory of the Shared Resources at the Fred Hutchinson Cancer Research Center (FHCRC) performed IHC to detect vWF+ cells in the lung biopsies. Lung biopsies were immediately fixed, processed, embedded in paraffin, and cut in 5 μm sections as described above. After epitope retrieval, slides were incubated with 3% hydrogen peroxide to block endogenous peroxidase activity, and then incubated in TBS buffer containing 0.25% (w/w) casein and 0.1% Tween 20 to block non-specific protein binding. They were incubated with anti-vWF antibodies (rabbit polyclonal, Dako, Glostrup, Denmark) or a concentration-matched control rabbit IgG as a negative control. Slides were washed, incubated with secondary antibodies (Mach2 rabbit HRP, Biocare Medical, Concord, CA) for 30 minutes, washed again, and then incubated further in DAB for 4 minutes twice. Nuclei were counter-stained with hematoxylin before being cover-slipped with Crystal mount. Cells were imaged as described above.

**Semi-quantitative reverse transcriptase-polymerase chain reaction (RT-PCR)**

Total RNA was purified using RNeasy spin column (Qiagen, Valencia, CA) and treated with RNase-free DNase (Qiagen) according to the manufacturer’s protocol. Samples were then reverse-transcribed into cDNA with an oligo dT_12-18_ primer and Superscript II reverse transcriptase (Invitrogen, Carlsbad, CA) by incubating for 2 hours at 42^o^C, followed by the termination of the reaction by heat inactivation (65^o^C, 20 minutes). Primers for dog transcripts are listed in Table S2

**Supplemental Figures and Tables**

**Table S1. Demographics of the dogs**

|  |  |  |  |  |
| --- | --- | --- | --- | --- |
| Recipient | Sex | Age at irradiation (Mo) | Weight at irradiation (Kg) | DRB allele |
|  |  |  |  |  |
| Dogs with DS-1 infusion | | |  |  |
| H303 | F | 15.9 | 13.0 | DRB1*006:01, DRB1*015:01 |
| H332 | M | 10.5 | 11.9 | DRB1*001:02, DRB1*006:01 |
| H336 | M | 15.5 | 11.0 | DRB1*006:01, DRB1*015:01 |
| H447 | F | 6.7 | 12.4 | DRB1*001:02, DRB1*009:01 |
| H450 | M | 8.6 | 18.5 | DRB1*006:01, DRB1*011:01 |
|  |  |  |  |  |
| Control dogs | |  |  |  |
| H065 | M | 23.7 | 15.7 | DRB1*008:01, DRB1*017:01 |
| H136 | M | 16.8 | 11.8 | DRB1*019:01, DRB1*024:01 |
| H181 | F | 11.9 | 10.4 | DRB1*009:01, DRB1*015:01 |
| H202 | F | 8.6 | 13.8 | DRB1*002:01, DRB1*002:01 |
| H233 | M | 8.9 | 10.5 | DRB1*006:01, DRB1*015:01 |
|  |  |  |  |  |
| Dog of DS1 origin | |  |  |  |
| G060 | F | (not irradiated) | (not irradiated) | DRB1*001:02, DRB1*006:01 |
|  |  |  |  |  |

| **Supplemental Table S2.** List of dog primers | |  |
| --- | --- | --- |
|  | 5' primer | 3' primer |
| Canine CD34 | CAGAAACCGTGATTACTCCTACCAC | AGCTCTAGGCAGATACCTTGGTTC |
| Canine CDH5/CD144/VE-cadherin | CACAGCCACAGTACTGGTCAACC | TGCGGATGGAATATCCAATGCTCC |
| Canine KDR | GAACTGAAGACAGGCTACTTGTCC | CTCTGACTACTGGTGATGCTGTCC |
| Canine vWF | GTCAGATTCAACCATCTTG GCCAC | GGGATGGTGGACATGACATAGCAC |
| Canine TEK/TIE2 | CTTTAAGATACAGCCTTTCCCATCC | CTGGTTCATTAAGGCTTCAAAGTCC |
| Canine PECAM1/CD31 | CTACCAAATCACCTCGAATGAAACC | TTCTGTGTATTCCACATCCAACGTC |
| Canine MCAM/CD146 | GGAGTCTCAGGAAGTCACTGTCC | CTTCAGGTTGTGTAACTGGAGCAC |
| Canine ACTB | GATGACGATATCGCTGCGCTTGTG | CATCACGATGCCAGTGGTGCGG |
| Canine PPIA/CYPA | CCGTGTTCTTTGACATCGCCGTG | GTTCAGATAAAACAGGAGTTAAGATTC |


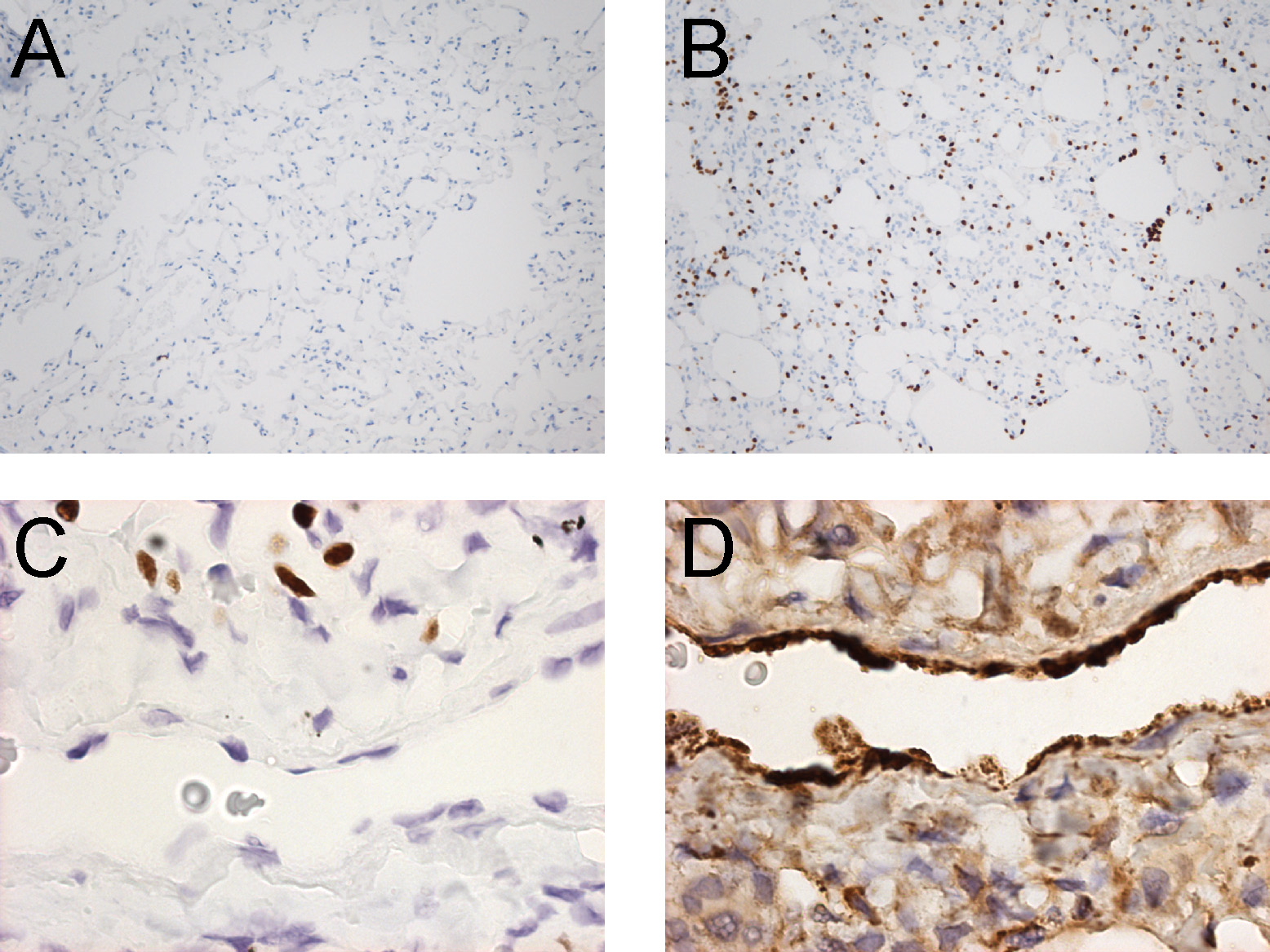


**Figure S1. Immune histochemistry of TTF1 and vWF in canine lung.** Panels A and B: Alveoli were incubated with concentration-matched rabbit IgG and anti-TTF1 antibodies, respectively. Bound antibodies were detected with HRP-conjugated secondary antibodies and DAB (brown staining). Nuclei were counter-stained with hematoxylin (blue). Original objective, X20. Panels C and D: Endothelial cells in lung capillary are negative for TTF1 but positive for vWF (Panels C and D, respectively). Original objective, X100.


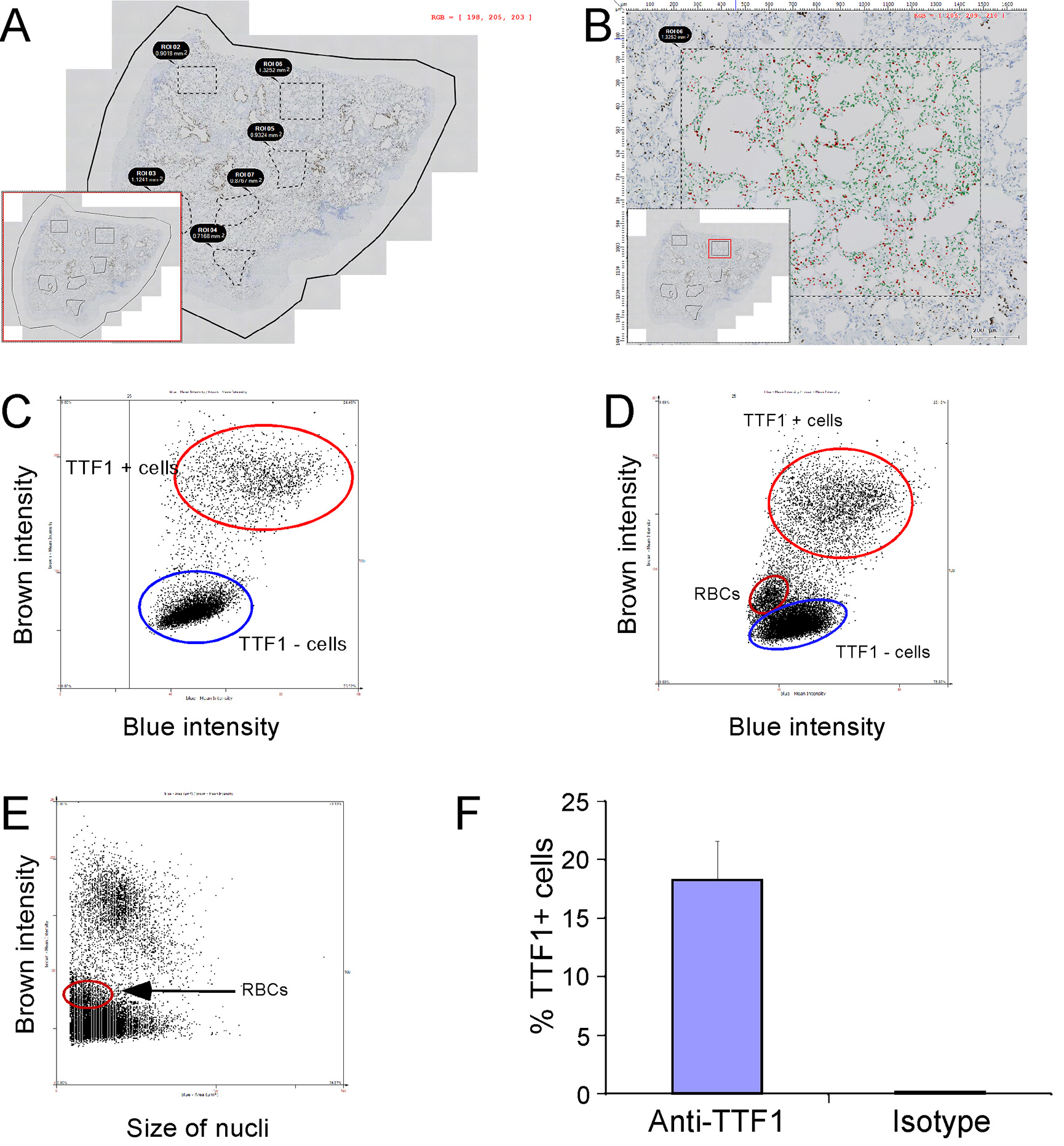


**Figure S2. Quantification of TTF-1 positive cells in immune histochemistry.** In order to quantify TTF-1+ cells in alveoli of DS1 treated and untreated dogs, HistoQuest software from Tissuegnostics (Vienna, Austria) was used. Panel A: Immune histochemistry of TTF-1 was conducted where brown and blue nuclei were TTF-1 positive and negative cells, respectively. Six areas on the section of alveoli were chosen blindly. Panel B: One of the six areas was selected (the red square in the insert), and color and size segmentations were applied to separate TTF-1+ and TTF-1 negative nuclei and to identify single cells. TTF-1 positive and negative cells were pseudo-colored in red and green, respectively. Panels C and D: Intensity of brown (TTF-1+ cells) and blue (TTF-1- cells) staining from non-irradiated control and irradiated lungs, respectively. Red blood cells (RBCs) were identified in the irradiated lung in Panel D. Size of RBCs was smaller than alveolar cells as expected (Panel E). Panel F: Immune histochemistry using anti-TTF1 antibodies and concentration-matched rabbit IgG (Isotype) was conducted. % of TTF1+ cells were calculated as a ratio of brown cell number over brown+blue cell number. The values are represented as an average of 3 sections (>6 areas per section).


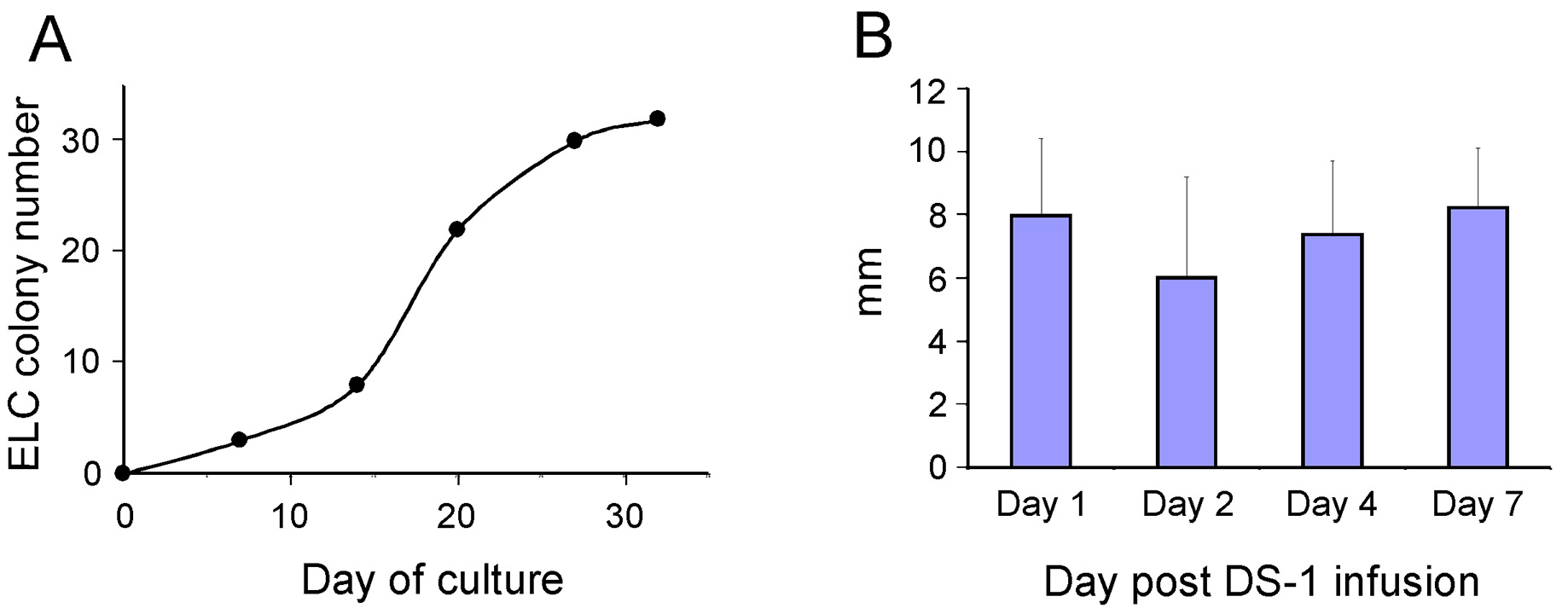


**Figure S3. Kinetics of ELC colony assay and growth of the colonies. Panel A:** PBMC (10×10^6^) from one of the DS1 dogs (H332) were harvested one day post DS-1 infusion, and cultured for 31 day as shown in X-axis. Number of ELC colonies bigger than 2 mm diameter was scored. **Panel B:** PBMC of the same dog (H332) were harvested 1 to 7 days post DS-1 infusion, and cultured for 30-31 days. Size of the colonies was measured (mm in diameter), and means+/-SDs were calculated (n=32, 2, 4, and 30 for Day 1, 2, 4, and 7, respectively).


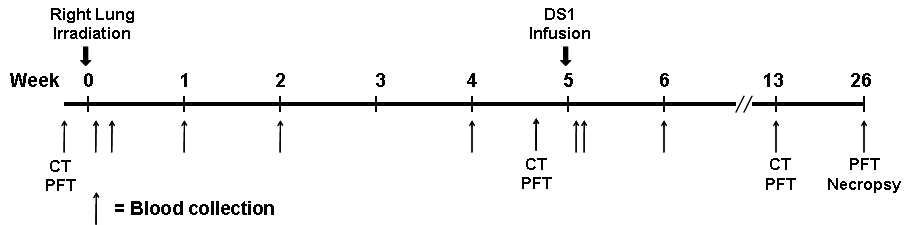


**Figure S4. Schematic diagram of experimental design for lung irradiation and DS1 cell infusion.**
